# Rebuttal from Gregory D. Funk and Alexander V. Gourine

**DOI:** 10.1113/JP276282

**Published:** 2018-06-26

**Authors:** Gregory D. Funk, Alexander V. Gourine

**Affiliations:** ^1^ Department of Physiology Neuroscience and Mental Health Institute Women and Children's Health Research Institute (WCHRI) Faculty of Medicine and Dentistry University of Alberta Edmonton Alberta Canada; ^2^ Centre for Cardiovascular and Metabolic Neuroscience, Neuroscience, Physiology & Pharmacology University College London London UK

Our colleague's thesis is that the work from Gourine, Funk and coworkers ‘does not provide conclusive evidence for their hypothesis of an involvement of astrocytes as central O_2_ sensors in the ventilatory response to hypoxia (HVR) especially in awake animals and humans’ (Teppema, 2018). While we agree that unequivocal evidence is not yet available, we emphasize that the converse is also true; there is no conclusive evidence against this hypothesis. The main counter point offered against contribution of a central, excitatory hypoxia‐sensing mechanism is that some of the experiments cited in support of the hypothesis are not perfect; that alternative explanations can be found. Indeed, few experiments considered in isolation are perfect, but available data considered *en masse* (Gourine *et al*. 2005; Angelova *et al*. 2015; Gourine & Funk, 2017; Rajani *et al*. 2018; Sheikhbahaei *et al*. 2018) strongly challenge the prevailing dogma that the entire hypoxia‐induced increase in ventilation originates from the carotid body (CB; or other peripheral chemoreceptor site).

To begin, many criticisms or alternative interpretations offered by our colleague appear to be based on the invalid assumption that astrocytes and their properties are similar throughout the CNS; e.g. he states ‘… given the CO_2_ sensitivity of astrocytes’; ‘that O_2_ sensitivity is a general property of astrocytes …’; and ‘… the resulting fall in P aC O2 would cause vasoconstriction …’ (Teppema, 2018). The view of astrocytes as a homogeneous cell population is not tenable (Turovsky *et al*. 2016; Chai *et al*. 2017). Even within the brainstem, retrotrapezoid and preBötzinger complex (preBötC) astrocytes differ markedly in morphology, protein expression and CO_2_/pH sensitivity (Gourine *et al*. 2010; Huckstepp *et al*. 2010a,*b*; Angelova *et al*. 2015; Sheikhbahaei *et al*. 2018). Three types of preBötC astrocytes have been distinguished based solely on electrophysiological properties (Grass *et al*. 2004).

Second, CB denervation studies are historically important, but, as discussed at length from both sides (Gourine & Funk, 2017; Funk & Gourine, 2018; Teppema, 2018), unlikely to ever be conclusive. Thus, the focus of our colleague on data from anaesthetized or conscious peripherally chemodenervated animals (Gourine *et al*. 2005; Angelova *et al*. 2015; Rajani *et al*. 2018) is not insightful. Experiments in dogs and goats demonstrate that isolated brain hypoxia facilitates breathing when CBs are normoxic/normocapnic, but not when CBs are denervated (Daristotle *et al*. 1991; Curran *et al*. 2000). These data indicate that expression of a central hypoxia sensing mechanism depends on tonic, but not necessarily chemosensory, drive from the CBs, and emphasize the ambiguity of data obtained in CB denervation studies.

The real arbiter of the physiological relevance of central hypoxia sensing mechanism is what happens when CBs remain intact while putative central oxygen sensing mechanisms are experimentally perturbed. Thus, key experimental studies (largely ignored by our colleague) are those in awake, CB‐intact animals in which blockade of astroglial vesicular release mechanisms or facilitation of ATP degradation by ectonucleotidase expression at the level of the preBötC consistently reduces the HVR (Angelova *et al*. 2015; Sheikhbahaei *et al*. 2018). This is seen also in anaesthetized CB intact or peripherally chemodenervated animals (Gourine *et al*. 2005; Angelova *et al*. 2015; Rajani *et al*. 2018). The results of these experiments cast serious doubt on the prevailing concept that the CB is the only respiratory oxygen sensor; the most parsimonious explanation is the existence of an excitatory astrocyte‐mediated CNS component of the hypoxic ventilatory response.

## Call for comments

Readers are invited to give their views on this and the accompanying CrossTalk articles in this issue by submitting a brief (250 word) comment. Comments may be submitted up to 6 weeks after publication of the article, at which point the discussion will close and the CrossTalk authors will be invited to submit a ‘Last Word’. Please email your comment, including a title and a declaration of interest, to jphysiol@physoc.org. Comments will be moderated and accepted comments will be published online only as ‘supporting information’ to the original debate articles once discussion has closed.

## Additional information

### Competing interests

None declared.
